# Systematic Review and Meta-Analysis of Candidate Gene Association Studies With Fracture Risk in Physically Active Participants

**DOI:** 10.3389/fgene.2020.00551

**Published:** 2020-06-16

**Authors:** Edward Ryan-Moore, Yiannis Mavrommatis, Mark Waldron

**Affiliations:** ^1^Faculty of Sport, Health and Applied Sciences, St Mary's University, London, United Kingdom; ^2^Fulham Football Club, Sports Science and Medicine, London, United Kingdom; ^3^Nell Health Ltd., Genetics & Nutrition, London, United Kingdom; ^4^Research Centre in Applied Sports, Technology, Exercise and Medicine, Swansea University, Swansea, United Kingdom; ^5^School of Science and Technology, University of New England, Armidale, NSW, Australia

**Keywords:** human genetics, injury, intrinsic risk factors, fracture, bone

## Abstract

**Background:** Fractures are common in physically active populations and genetic differences may mediate injury risk.

**Objective:** To meta-analyse the pooled results of candidate gene association studies with non-osteoporotic fracture risk in physically active humans.

**Methods:** Systematic searching of databases returned 11 eligible studies published in English. Pooled odds ratios (ORs) with 95% confidence intervals (CI) were produced using allele contrast, recessive and homozygote contrast meta-analysis models to evaluate associations of risk alleles in the *COL1A1* (rs1800012), *COL2A1* (rs412777), *CTR* (rs1801197), *ESR1* (rs2234693 and rs9340799) *LRP5* (rs3736228), *VDR* (rs10735810, rs7975232, rs1544410, and rs731236) genes with fracture incidence.

**Results:** Eligible study quality was generally low (7/11) and no significant overall effect was found for any genetic variant with any comparison model (*p* > 0.05). A *trivial* reduction in fracture risk was found for female participants with the *COL1A1* Sp1 (rs1800012) T allele (*OR* = 0.48, 95% CI = 0.25–0.91, *p* = 0.03, *d* = –0.18).

**Conclusions:** No overall effect was found from the pooled results of included genetic variants on fracture risk in physically active participants. The *COL1A1* Sp1 rs1800012 T allele may reduce fracture risk in physically active females but further high-quality research with sex-specific analysis is required.

**Trial Registration:** (PROSPERO; CRD42018115008).

## Introduction

Fractures are major musculoskeletal injuries, accounting for 22% of all sport and recreation related injuries in the United States (Conn et al., 2003). Fracture rehabilitation requires substantial time away from competition/work for physically active populations, such as athletes or military personnel (Kaufman et al., 2000; Le Gall et al., 2006) and has a negative impact on performance (Hägglund et al., 2013). Fractures occur when exposure to extrinsic aetiological factors result in force transfer to bone, which exceeds the threshold tolerance of an individual (Meeuwisse et al., 2007) and may occur from acute impact forces or repeated loading with insufficient recovery (i.e., stress fractures; Bennell et al., 1999). Physical activity provides an important stimulus for bone health and is recommended to protect against osteoporotic fracture (Kohrt et al., 2004). However, this stimulus also represent an exposure to potentially injurious, forceful impact or repeated loading of the musculoskeletal system (Launay, 2015; Meardon et al., 2015; Bacon and Mauger, 2017; Schuh-Renner et al., 2017) which cause non-osteoporotic fractures which are the focus of the present meta-analysis.

Genetic differences have been shown to influence the inter-individual variability in fracture risk (Efstathiadou et al., 2001; Mann et al., 2001; Ji et al., 2010; Trajanoska et al., 2018) with heritable factors associated with between 20 and 54% of fracture liability depending on site and age (Andrew et al., 2004; Michaëlsson et al., 2005). Fracture risk is a complex trait, influenced by the cumulative effects of a currently unknown number of genetic variants, which interact to produce slight alterations in tissue composition, structure and regulation (Baumert et al., 2016; Kozlovskaia et al., 2017; Herbert et al., 2018). Genome wide association studies (GWAS) have identified single nucleotide polymorphisms (SNPs) which influence fracture risk in genes involved in skeletal structure and homeostasis via alterations in bone mineral density (Trajanoska et al., 2018). To confirm the findings of GWAS, or to identify novel genetic variants, contributing to variability in fracture risk, genetic association studies may select candidate genes, based on their mechanistic effect on fracture risk. SNPs can change the physiological functionality of a genetic product by altering the amino acid sequence or moderating expression directly. Others may not directly influence fracture risk but are frequently inherited, or in linkage disequilibrium, with unidentified variants that do. The major structural protein of bone is type 1 collagen (Mann et al., 2001), whilst vitamin D is also fundamental for bone homeostasis (DeLuca, 2005). SNPs in the collagen type 1 alpha 1 (*COL1A1*), vitamin D receptor (*VDR*), and LDL receptor related protein 5 (LRP5) genes have been associated with a three- to eight-fold increase of fracture risk among physically active participants in some studies (Chatzipapas et al., 2009; Blades et al., 2010; Korvala et al., 2010), yet others have shown no association with the same SNPs (Cosman et al., 2013; Varley et al., 2018). Several genetic variants within the *COL1A1, LRP5*, and *VDR* genes, along with other candidate genes, have been inconsistently associated with fracture risk (Korvala et al., 2010; Varley et al., 2018). Researchers exploring genetic association with fracture risk often combine male and female participants, thus improving the statistical power of the analysis. It can be argued that autosomal (i.e., non-sex-specific) genes may be compared equivalently between the sexes. However, physically active females have a significantly greater incidence and absolute risk of fracture compared to males (Kaufman et al., 2000; Wentz et al., 2011; Waterman et al., 2016), which may influence the relative contribution of genetic differences to fracture risk. Therefore, combining physically active male and female participants in genetic association with fracture risk may contribute to the inconsistency observed across studies.

A meta-analysis of 370 studies found statistically significant heterogeneity in 14 out of 36 groups of genetic association studies on the same topic with stronger effects in the first study of a topic than subsequent replication attempts in 25 cases (Ioannidis et al., 2001). This may result from spurious findings which are not validated in subsequent research or because a gene effect may be stronger in some sub-populations than others (Ioannidis et al., 2001). Potential limitations such as linkage disequilibrium, population stratification and Hardy-Weinberg equilibrium (HWE) are inherent in genetic association studies, contributing to study heterogeneity (Ioannidis et al., 2003; Salanti et al., 2005). Additional variation resulting from issues relating to study design and quality, such as sample size calculations and reporting of participant characteristics are inconsistent in genetic association studies of fracture risk (Välimäki et al., 2005; Suuriniemi et al., 2006; Chatzipapas et al., 2009; Yanovich et al., 2012; Varley et al., 2016), yet omission of methodological details such as these can have a substantial influence on the study outcome (Ioannidis et al., 2003). Therefore, an independent analysis of study quality is necessary to understand the limitations of published genetic association studies in the extant literature.

Meta-analyses pool results from individual genetic association studies to evaluate overall effects with greater statistical power and identify heterogeneity between studies (Ioannidis et al., 2001; Salanti et al., 2005). It is unclear which genetic variants are consistently associated with fracture risk and whether the magnitude of the effect is dependent on factors such as gender or study quality. Therefore, the aim of this systematic review and meta-analysis was to evaluate the findings and quality of genetic association studies with non-osteoporotic fracture risk in physically active humans with sub-analysis of the influence of gender on overall findings.

## Methods

### Search Strategy

The current review is registered on the PROSPERO International prospective register of systematic reviews (Trial Registration: CRD42018115008) and was conducted in accordance with the Preferred Reporting Items for Systematic Reviews and Meta-Analyses (PRISMA) statement guidelines (Moher et al., 2009). A literature search to identify articles evaluating the association of genetic variants with fracture injury incidence was completed using a pre-determined search strategy in the PubMed, SPORTDiscus (EBSCO) and Science Direct databases from their inception on the 30th of October 2018. The exact search terms used were: Fracture OR Fractures AND Gene OR Allele OR Polymorphism OR SNP OR Variant OR Genetic. The title and abstract of search results were screened for relevant articles, which were selected for full text evaluation by two authors independently (ERM and MW) using predetermined eligibility criteria. The reference list of eligible articles was subsequently screened for further articles.

### Inclusion and Exclusion Criteria

Genetic case-control association studies of fracture occurrence in physically active humans published in English in a scientific peer-reviewed journal were included in the analysis to identify previously investigated genetic variants. Participants were required to be healthy, and clearly reported as at least moderately physically active, as part of either their occupation (e.g., athletes and military personnel) or lifestyle, as defined by the ACSM's Guidelines for Exercise Testing and Prescription (Thompson et al., 2013). Any case studies or association studies with osteoporotic fracture, osteogenesis imperfecta, fracture recovery, and genetic risk score evaluation studies were excluded.

### Study Selection and Data Extraction

Following the removal of duplicates, studies were screened independently by two reviewers (ERM and MW) with discrepancies concluded by consensus agreement. The following data were extracted from eligible articles: (1) study details (author, publication date, country of origin); (2) population characteristics (gender, age, ethnicity, physical activity); (3) genetic variant(s). Quality assessment and risk of bias assessments were carried out using the Q-Genie (Sohani et al., 2015) and modified ROBINS-I (Sterne et al., 2016; Qasim et al., 2019) tools independently by two authors (ERM and YM). The Q-Genie tool categorizes studies as either poor, moderate, or good quality with the modified ROBINS-I determining risk of bias as low, moderate, serious, or critical. Study characteristics data are presented as means ± standard deviations.

### Meta-Analysis

Data analysis was performed by one author (ERM) and reviewed by another (MW). Data were extracted, where possible, in the form of genotype frequency distributions between fractures (cases) and non-fractures (controls) for males, females, and combined if not reported separately. If only percentage distributions were reported, participant number for each group was calculated using overall participant number. If neither of the above was possible, authors were contacted directly for data.

**Figure 1:**
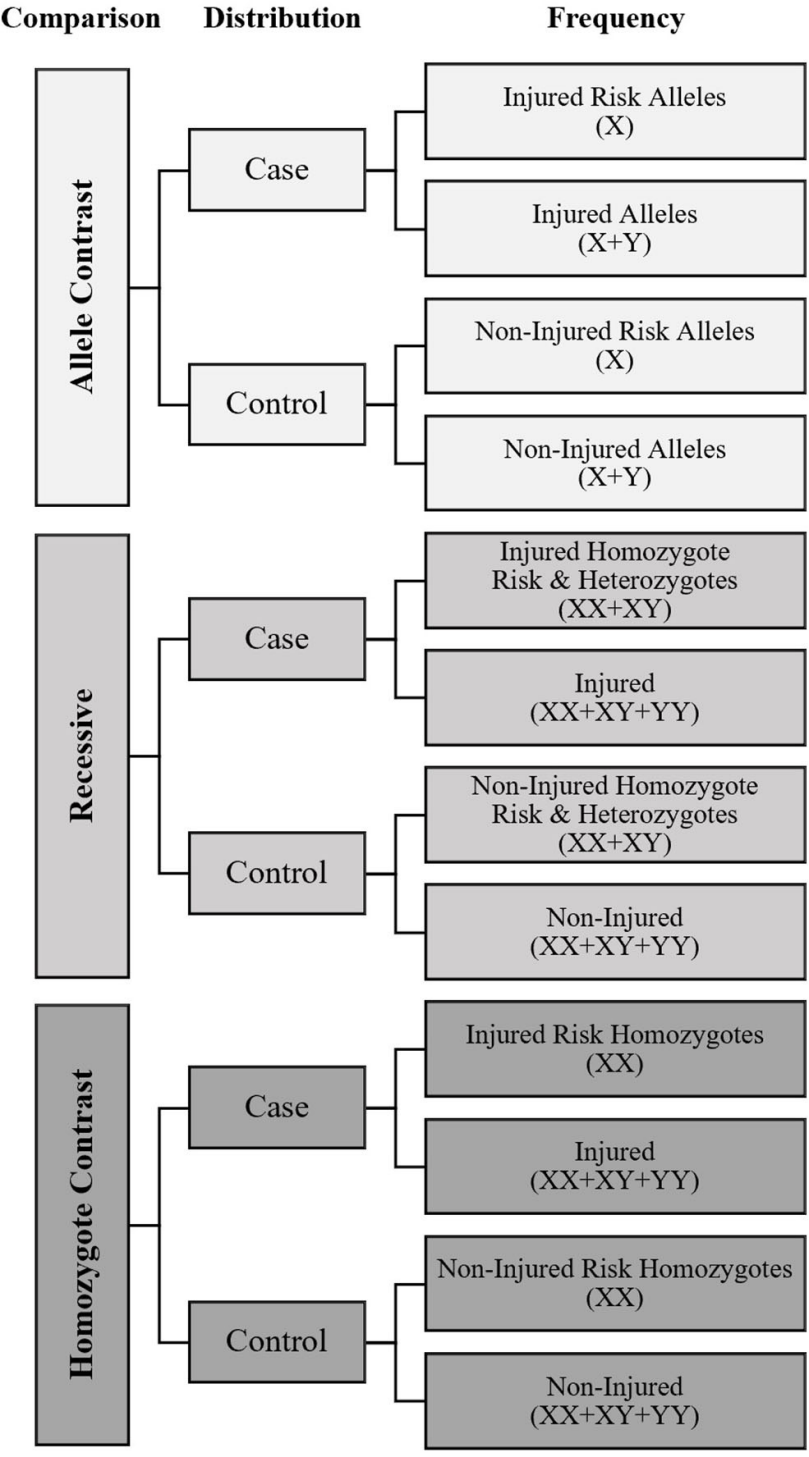
Meta-Analysis data input diagram*. X*, Risk Allele of genetic variant for fracture as defined by mechanistic rationale or candidate gene association study; *Y*, Non-Risk Allele of genetic variant. Symbols in parentheses indicate how the frequency counts were calculated to establish if differences in the risk allele distribution were present between cases and controls for each model.

A meta-analysis was performed to calculate overall fracture risk, with sub-analysis of males, and females separately, as odds ratios (OR) for each SNP, with extracted data available from two or more studies using the following genetic association meta-analysis models of comparison: allele contrast, recessive, and homozygote contrast, as recommended by Lee (2015). The frequency distribution between fracture cases with the candidate risk allele, as theoretically identified by the studies, and non-injured controls was entered into a dichotomous Mantel-Haenszel meta-analysis for each model as shown in Figure 1 using RevMan 5.3 software (Cochrane Collaboration, Oxford, United Kingdom) to generate pooled ORs with 95% confidence intervals (CI).

Genetic models were analyzed using either fixed (*I*^2^ <20%) or random (*I*^2^ ≥ 20%) effects models, depending on heterogeneity between studies, quantified with the *I*^2^ statistic with sub-analysis of sex. To provide a qualitative indication of the magnitude of effect observed, the OR produced by meta-analysis were converted to the standard mean difference and described in line with those suggested for Cohen's d (Cohen, 1988; Sánchez-Meca et al., 2003). Funnel plots were generated using the outcome of all included SNPs for each genetic comparison models to allow visual interpretation of potential biases (Sterne et al., 2011). To evaluate the potential of bias between studies, Egger's Test (Egger et al., 1997) was conducted to indicate the presence of funnel plot asymmetry using SPSS (IBM SPSS Statistics for Windows, IBM Corp, Version 24.0., Armonk, NY, United States) for each of the genetic comparison models.

## Results

### Study Selection

**Figure 2:**
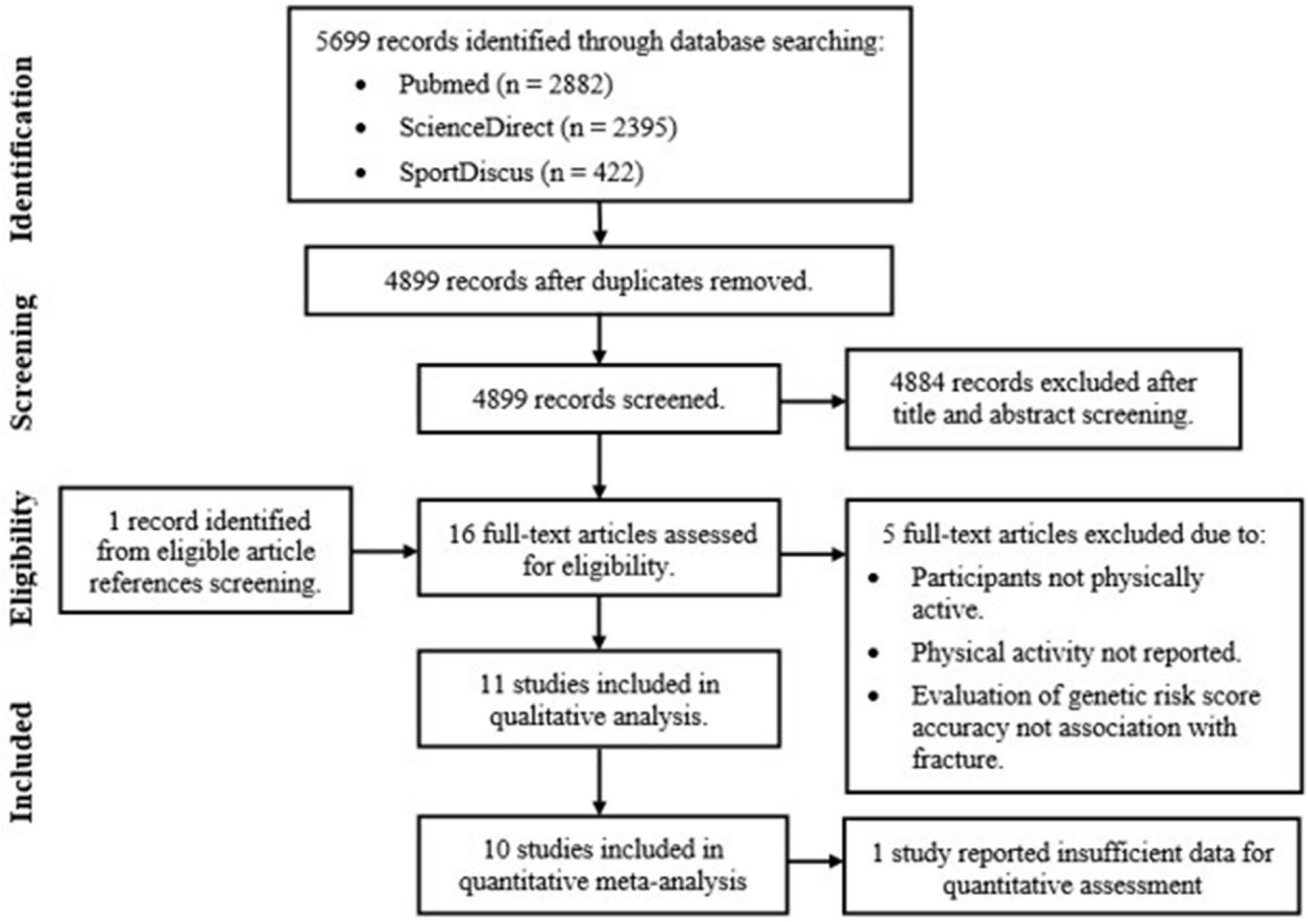
Genetic case-control association study of fracture risk in physically active participant systematic review and meta-analysis study selection process.

Figure 2 outlines the results of the study selection process. Once duplicates were removed, reviews, case-studies and abstracts were excluded along with studies investigating clinical populations (including osteogenesis imperfecta or osteoporotic fractures patients) and fracture recovery. Reference list screening of the remaining articles provided one additional study, resulting in the full text review of 16 eligible studies. Five articles were excluded based on predetermined inclusion criteria, with qualitative assessments completed on the remaining 11 articles. Only 10 articles were included in the final meta-analysis. This was due to lack of reported or available data in one study, determined after contacting Yanovich et al. (2012).

### Study Characteristics

The characteristics of each included study are summarized in Table 1. A total of 39 SNPs from 14 different genes were analyzed at least once in the included studies. The mean sample of the studies was 499 ± 385 (males: 454 ± 400 and females: 117 ± 72). However, a convenience sample of the same 501 elite athletes from various sports (433 males and 68 females) was replicated in three studies evaluating different genetic variants with facture risk (Varley et al., 2015, 2016, 2018). Excluding these duplications, a total of 4,462 (3,676 males and 686 females) different physically active participants of various nationalities and ethnicities, aged 4–32 years are included. Of these, 961 were classified as fracture cases (779 males and 182 females) and 3,501 considered non-fracture controls (2,997 males and 504 females).

**Table 1:** Summary of articles identified from systematic review of genetic case-control association studies with fracture risk in physically active participants.

**References**	**Gene (SNPs)**	**Participant characteristics**					**Result**
		**Sample**	**Case/Control and sex**	**Physical activity**	**Age (y)**	**Ethnicity**	
Blades et al. (2010)	*COL1A1*: (rs1800012) *COL1A2*: (rs412777)	M and F English Children presented to A&E following impact trauma	Fracture = 197-(*M =* 124, *F =* 73) Control = 187-(*M* = 106, *F =* 81) TOTAL = 384-(*M =* 230, *F =* 154)	Recreational physical activity	*M =* 11 ± 3 *F =* 11 ± 3 (4–16)	Caucasian	*COL1A2* “PP” genotype halved fracture risk (*p* = 0.01, *OR* = 0.45, 95% CI = 0.24–0.82). *COL1A1* “s” allele trebled fracture risk in pre-pubertal children (*p* = 0.004, *OR* = 3.1, 95% CI = 1.43–6.63)
Chatzipapas et al. (2009)	*VDR*: (rs10735810, rs1544410, rs731236, rs7975232)	M only Military personnel	Stress Fracture = 32 Control = 32 TOTAL = 64	Military Duties	23 ± 3 (19–30)	Unknown	*VDR* rs10735810 “f” (*p* = 0.017, *OR* = 2.8, 95% CI = 1.2–6.3) and possibly rs1544410 “B” (*p* = 0.051, *OR* = 2.2, 95% CI = 1.0–4.4) alleles increase stress fracture risk
Cosman et al. (2013)	*COL1A1*: (rs1800012) *ESR1*: (rs2234693, rs9340799) *VDR*: (rs1544410)	M and F US Military Recruits	Stress Fracture = 69-(*M =* 43, *F =* 26) Control = 822-(*M =* 712, *F =* 110) TOTAL = 891-(*M =* 755, *F =* 136)	Basic Military Training	*M =* 19 ± 1 *F =* 18 ± 1 (18–20)	M: 86.5% Caucasian, 5% Asian, 8.5% Black F: 79.4% Caucasian, 11% Asian, 9.6% Black	No genetic association with stress fracture incidence (*p* > 0.05)
Korvala et al. (2010)	*COL1A1*: (rs1800012, rs2696247, rs2586488, rs406226) *COL1A2*: (rs2301643, rs3216902, rs406226) *CTR*: (rs1801197) *IL-6*: (rs1800795) *LRP5*: (rs2277268, rs4988321, rs556442, rs3736228) *VDR*: (rs10735810, rs1544410, rs731236)	M only Finnish Military Conscripts	Stress Fracture = 72 Control = 120 TOTAL = 192	Basic Military Training	*M =* 20 ± 2 (18–27)	Unknown	Absence of CTR C allele and/or VDR C-A haplotype increased stress fracture risk (*p* = 0.007, *OR* = 3.22, 95% CI = 1.38–7.49). LRP5 haplotype A-G-G-C increased stress fracture risk (*p* = 0.031, *OR* = 2.72, 95% CI = 1.10–6.73) increasing when combined with the VDR C-A haplotype (*p* = 0.028, *OR* = 3.85, 95% CI = 1.16–12.84) but was mediated by body mass and BMI
Suuriniemi et al. (2003)	*COL1A2*: (rs412777)	F Finnish children	Fracture = 37 Control = 221 TOTAL = 258	2.8–3.0 h/week	*F =* 11 ± 1 (10–12)	Unknown	COL1A2 P allele (either PP or Pp genotype) increased fracture risk compared to pp genotype (*p* = 0.007, *OR* = 4.1, 95% CI = 1.4–12.4)
Välimäki et al. (2005)	*ESR1*: (rs2234693, rs9340799)	M Finnish Military Conscripts	Stress Fracture = 15 Control = 164 TOTAL = 179	Basic Military Training	*M =* 19 ± 1 (18–20)	Unknown	No genetic association with stress fracture incidence (*p* > 0.23)
Varley et al. (2015)	*TNFSF11*: (rs1021188, rs9594738) *TNFRSF11A*: (rs3018362) *TNFRSF11B*: (rs4355801)	M and F Elite Athletes from USA and UK (SFEA Cohort)	Stress Fracture = 125-(*M =* 98, *F =* 27) Control = 376-(*M =* 335, *F =* 41) TOTAL = 501-(*M =* 433, *F =* 68)	Professional Athletes of Various Sports	Stress Fracture = 27.2 ± 6.9 Control = 24.2 ± 5.5	Caucasian: Stress Fractures 83.2%, Controls 79.9% Other Unknown: Stress Fracture 16.8%, Controls 20.1%	TNFSF11 rs1021188 AA (*p* = 0.024, *OR* = 2.9, 95% CI = 1.2–7.3) and TNFRSF11A rs3018362 GA+AA (*p* = 0.049, *OR* = 1.5, 95% CI = 1.0–2.4) individuals showed increased risk of stress fracture in comparison to GG individuals
Varley et al. (2016)	*P2X7R*: (rs1653624, rs3751143, rs2230912, rs2230911, rs1718119, rs28360457, rs7958316, rs7958311, rs208294, rs28360447, rs17525809, rs35933842)	M and F Israeli Defence Force Soldiers and Elite Athletes from USA and UK (SFEA Cohort)	Military = 210-(*M =* 198, *F =* 12), Stress Fracture = 43-(*M =* 41, *F =* 2) Control = 167-(M 157, *F =* 10) Elite Athletes = 501-(*M =* 433, *F =* 68) Stress Fracture = 125-(*M =* 98, *F =* 27) Control = 376-(*M =* 335, *F =* 41) TOTAL = 711-(*M =* 631, *F =* 80)	Military Training and Professional Athletes of Various Sports	Military: Stress Fracture = 20.3 ± 1.6, Control = 18.9 ± 0.5 Athletes: Stress Fracture = 27.7 ± 7.5, Control = 24.4 ± 5.4	Elite Athletes: Stress fractures 83.2% Caucasian, 16.8% other. Controls 79.9%, Caucasian, 20.1% other Military: Stress Fracture 36% non-Ashkenazi, 64% Ashkenazi. Control, 45% non-Ashkenazi, and 55% Ashkenazi	P2X7R rs1718119 A allele (*p* = 0.01) and rs3751143 C allele (M only) (*p* = 0.04) associated with stress fracture occurrence in military participants. P2X7R rs3751143 C allele associated with stress fracture occurrence (*p* = 0.05) in elite athletes. After correcting for multiple comparisons using the false discovery rate test none of the findings remained significant (*p* > 0.05)
Varley et al. (2018)	*COL1A1*: (rs1800012) *CTR*: (rs1801197) *GC*: (rs4588, rs7041) *LRP5*: (rs3736228) *SOST*: (rs1877632) *VDR*: (rs10735810, rs7975232, rs731236, rs1544410) *WNT16*: (rs3801387)	M and F Elite Athletes from USA and UK (SFEA Cohort)	Stress Fracture = 125-(*M =* 98, *F =* 27) Control = 376-(*M =* 335, *F =* 41) TOTAL = 501-(*M =* 433, *F =* 68)	Professional Athletes of Various Sports	Stress Fracture = 27.7 ± 7.5 Control = 24.4 ± 5.4	Caucasian: Stress Fractures 83.2%, Controls 79.9% Other Unknown: Stress Fracture 16.8%, Controls 20.1%	SOST rs1877632 TT+TC v CC (*p* = 0.02), VDR rs10735810 (*p* = 0.01), and rs731236 (*p* = 0.01) C homozygotes (both M only) were associated with stress fracture occurrence. After correcting for multiple comparisons using the false discovery rate test none of the findings remained significant (*p* > 0.05).
Yanovich et al. (2012)	*ANKH*: (rs4701616) *CALCR*: (rs12154667, rs1548456) *CBG*: (rs11629171, rs2281518) *COL1A2*: (rs420257, rs42517, rs42522, rs24531, rs413826) *IL6*: (rs1554606) *LRP4*: (rs2306033) *NR3C1*: (rs4244032, rs12656106) *ROR2*: (rs10992075) *VDR*: (rs4328262) Additional Not Reported	M and F Israeli Defence Force Soldiers	Stress Fracture = 182-(*M =* 165, *F =* 17) Control = 203-(*M =* 162, *F =* 41) TOTAL = 385-(*M =* 327, *F =* 58)	Military Training	Stress Fracture = 20.1 ± 1.7 (18–32) Control = 20.2 ± 1.3 (18–32)	Ashkenazi 49.5%, Non-Ashkenazi 38.1%, and Unknown 12.4%	NR3C1, ANKH, VDR, ROR2, CALCR, IL6, CBG, and COL1A2 associated with increased risk of stress fracture (*p* < 0.05). NR3C1, AR, VDR, CALCR, COL1A2, and LRP4 associated with reduced risk of stress fracture (*p* < 0.05). After correcting for multiple comparisons using the false discovery rate test none of the findings remained significant (*p* > 0.05)
Zhao et al. (2016)	*GDF5*: (rs143383)	M Chinese Military Recruits	Stress Fracture = 189 Control = 1209 TOTAL = 1398	Basic Military Training	Stress Fracture = 18.5 ± 1.4 Control = 18.5 ± 1.8	Unknown	GDF5 rs143383 T allele (*p* < 0.001, *OR* = 1.8, 95% CI = 1.4–2.3) and TT genotype (*p* = 0.002, *OR* = 1.8, 95% CI = 1.3–2.5) increased risk of stress fracture occurrence in comparison to C allele and TC+CC genotypes, respectively

*M, male; F, female; SNP, single nucleotide polymorphism; OR, odds ratio; CI, confidence interval*.

Two studies focused on acute fracture risk in children (Suuriniemi et al., 2003; Blades et al., 2010), the other nine evaluated stress fracture risk in professional adult military and/or athlete groups. Only one study investigated female participants alone (Suuriniemi et al., 2003), four investigated only males (Välimäki et al., 2005; Chatzipapas et al., 2009; Korvala et al., 2010; Zhao et al., 2016), five included both combined and separate analysis for male and female participants (Yanovich et al., 2012; Cosman et al., 2013; Varley et al., 2015, 2016, 2018) with one reporting only pooled results for males and females (Blades et al., 2010).

**Figure 3:**
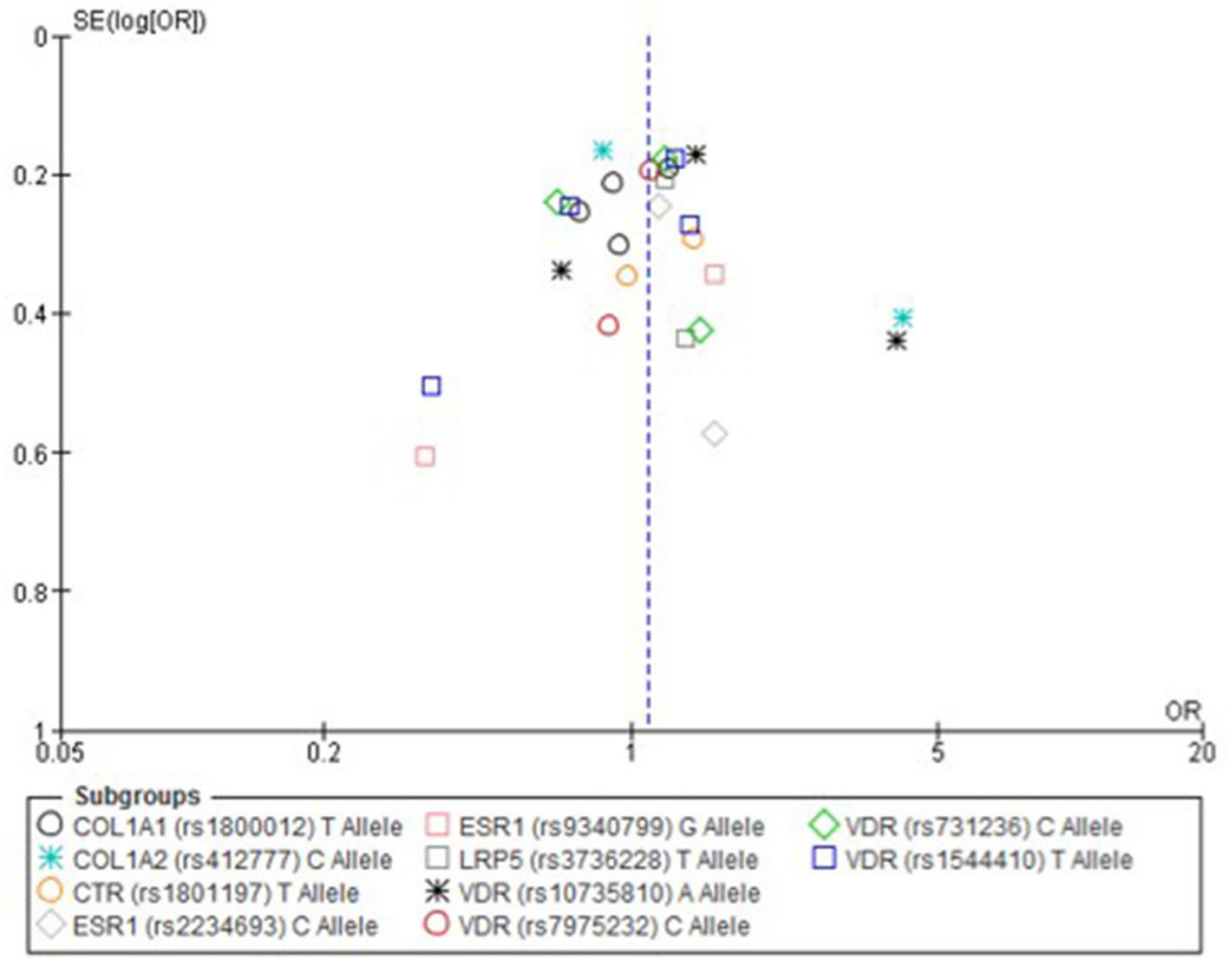
Funnel plot of single nucleotide polymorphisms replicated in studies investigating genetic association with fracture risk in physically active participants using the allele contrast model.

**Figure 4:**
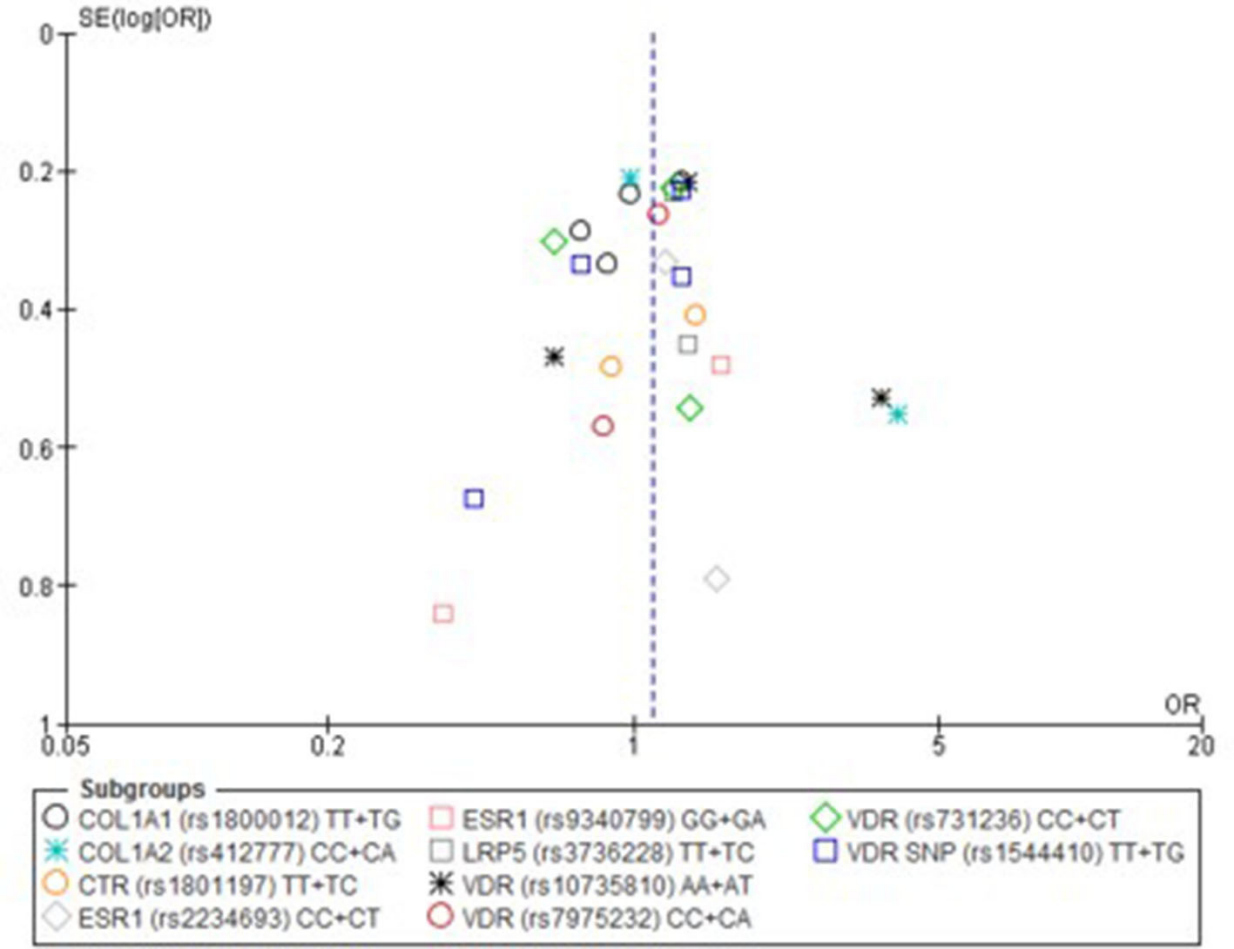
Funnel plot of single nucleotide polymorphisms replicated in studies investigating genetic association with fracture risk in physically active participants using the recessive model.

**Figure 5:**
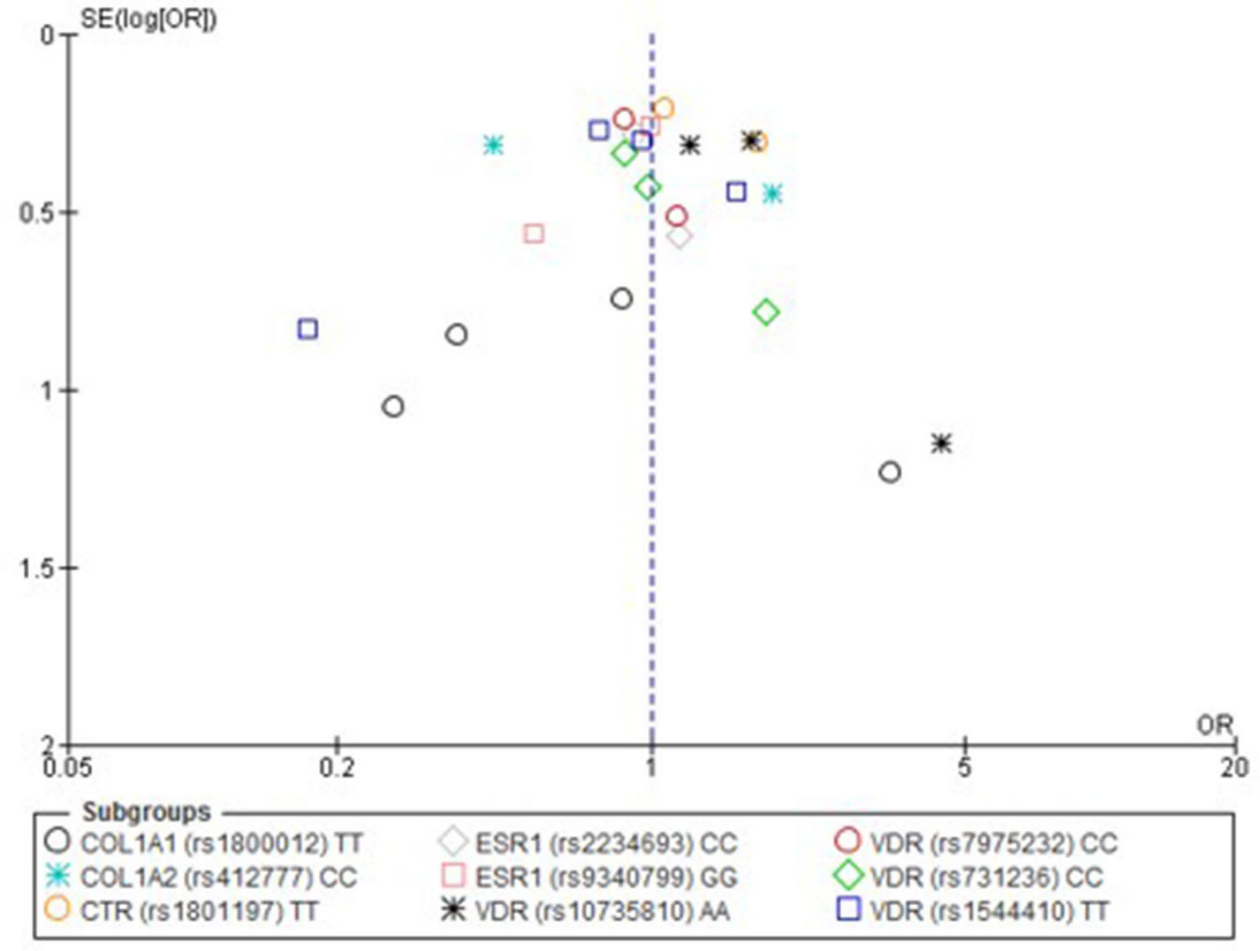
Funnel plot of single nucleotide polymorphisms replicated in studies investigating genetic association with fracture risk in physically active participants using the homozygote contrast model.

Only one paper achieved the highest classification of study quality (Blades et al., 2010), three were classified as moderate (Korvala et al., 2010; Cosman et al., 2013; Zhao et al., 2016), resulting in seven of the eligible studies defined as poor quality genetic association studies (Suuriniemi et al., 2003; Välimäki et al., 2005; Chatzipapas et al., 2009; Yanovich et al., 2012; Varley et al., 2015, 2016, 2018). The overall risk of bias judgement varied from moderate to critical and was predominantly affected by bias due to confounding and participant selection. A summary of the assessment, including domain level judgments, are presented in Table 2. The funnel plots for the allele, recessive and homozygote comparison models, shown in Figures 3–5, respectively, did not display a perfect funnel shape, but indicated no clear publication bias. The funnel plots generally display a cluster of large studies around the summary estimate and a lack of smaller studies spread beneath. The nine studies included in the Egger's test of this meta-analysis was just under the ten recommended as a rule of thumb for sufficient power by Sterne et al. (2011). However, the results of Egger's test indicated no sign of funnel plot asymmetry in any of the genetic meta-analysis comparison models, suggesting that there was no between-study bias within the included studies (*p* > 0.24).

**Table 2:** Risk of bias assessment judgements for genetic case-control association studies with fracture risk in physically active participants.

**References**	**Selection bias**	**Bias due to confounding**	**Bias in classification of exposure**	**Bias in assessment of outcome**	**Bias due to missing data**	**Bias in selection of reported results**	**Overall risk of bias**
Blades et al. (2010)	Moderate	Serious	Moderate	Moderate	Low	Moderate	Serious
Chatzipapas et al. (2009)	Serious	Serious	Serious	Serious	Low	Moderate	Serious
Cosman et al. (2013)	Moderate	Moderate	Low	Low	Low	Moderate	Moderate
Korvala et al. (2010)	Serious	Serious	Serious	Serious	Moderate	Moderate	Serious
Suuriniemi et al. (2003)	Moderate	Moderate	Moderate	Moderate	Low	Moderate	Moderate
Välimäki et al. (2005)	Moderate	Moderate	Moderate	Moderate	Low	Moderate	Moderate
Varley et al. (2015)	Serious	Moderate	Low	Moderate	Low	Serious	Serious
Varley et al. (2016)	Serious	Serious	Serious	Moderate	Serious	Serious	Serious
Varley et al. (2018)	Serious	Serious	Serious	Moderate	Low	Serious	Serious
Yanovich et al. (2012)	Serious	Serious	Serious	Low	Serious	Critical	Critical
Zhao et al. (2016)	Moderate	Moderate	Low	Low	Low	Low	Moderate

### Meta-Analysis

Ten genetic variants from six different genes; *COL1A1* (rs1800012), *COL2A1* (rs412777), *CTR* (rs1801197), *ESR1* (rs2234693 and rs9340799), *LRP5* (rs3736228), and *VDR* (rs10735810, rs7975232, rs731236, and rs1544410) were replicated at least once in seven of the 10 eligible studies, which constituted the quantitative meta-analysis. The summary statistics for each genetic comparison model meta-analysis are presented in Tables 3–6. Table 3 includes pooled analysis for all participants (male and females) of included studies, while Table 4 includes males and females from studies classified as good or moderate quality only. No statistically significant overall effect was found from the meta-analyses of any genetic model or SNP (*p* > 0.06). Tables 5, 6 include summary statistics of male and female only sub-group analysis, respectively. Sub-group analysis identified a significant reduction of fracture risk in female participants, with the T allele of the *COL1A1* rs1800012 SNP using the allele contrast model (*OR* = 0.48, 95% CI = 0.25–0.91, *p* = 0.03, *d* = –0.18), however this was not statistically significant in the recessive model (*OR* = 0.51, 95% CI = 0.24–1.06, *p* = 0.07, *d* = –0.16).

**Table 3:** Summary effects from the overall analyses of case-control association studies for genetic variants associated with fracture occurrence risk in physically active participants including all studies and sex sub-groups.

**Genetic variant and risk comparison model**	**Sample size**		**Test of heterogeneity**				**Test of overall association**		
	**Participants**	**Studies**	**Overall**		**Between sub-groups**		**FE/RE**	**OR (95% CI)**	***P***
	**Fracture/Control (risk model frequency)**		***I*^**2**^**	***P***	***I*^**2**^**	***P***			
***COL1A1*** **Sp1(rs1800012)**									
Allele Contrast: T	772 (20%)/2514 (20%)	4	17%	0.30	**67%**	**0.05**	FE	0.95 (0.76–1.19)	0.66
Recessive: TT+TG	455 (32%)/1465 (31%)	4	1%	0.41	57%	0.10	FE	0.99 (0.77–1.27)	0.91
Homozygote Contrast: TT	455 (1.5%)/1465 (3%)	4	0%	0.70	0%	0.63	FE	0.58 (0.25–1.32)	0.19
***COL1A2*** **PvuII (rs412777)**									
Allele Contrast: C	342 (49%)/607 (51%)	2	**92%**	**<0.001**	**92%**	**<0.001**	RE	1.81 (0.39–8.52)	0.45
Recessive: CC+CA	229 (62%)/393 (62%)	2	**83%**	**0.02**	**82%**	**0.02**	RE	1.81 (0.46–7.17)	0.40
Homozygote Contrast: CC	229 (11%)/393 (16%)	2	**85%**	**0.009**	**85%**	**0.009**	RE	0.87 (0.22–3.52)	0.85
***CTR*** **(rs1801197)**									
Allele Contrast: T	313 (90%)/767 (88%)	3	0%	0.56	0%	0.88	FE	1.27 (0.82–1.97)	0.29
Recessive: TT+TC	189 (92%)/477 (91%)	3	0%	0.68	0%	0.96	FE	1.23 (0.67–2.27)	0.51
Homozygote Contrast: TT	189 (57%)/477 (51%)	3	25%	0.26	38%	0.20	RE	1.23 (0.81–1.87)	0.33
***ESR1*** **PvuII (rs2234693)**									
Allele Contrast: C	119 (76%)/1455 (73%)	2	0%	0.41	44%	0.18	FE	1.25 (0.80–1.95)	0.33
Recessive: CC+CT	80 (83%)/953 (79%)	2	0%	0.79	0%	0.49	FE	1.28 (0.69–2.35)	0.43
Homozygote Contrast: CC	80 (31%)/953 (32%)	2	48%	0.14	**73%**	**0.05**	RE	0.92 (0.43–1.98)	0.84
***ESR1*** **XbaI (rs9340799)**									
Allele Contrast: G	124 (89%)/1519 (86%)	2	**68%**	**0.04**	0%	0.81	RE	1.02 (0.35–3.00)	0.96
Recessive: GG+GA	80 (91%)/953 (90%)	2	33%	0.23	0%	0.80	RE	1.11 (0.40–3.09)	0.85
Homozygote Contrast: GG	80 (46%)/953 (49%)	2	4%	0.35	0%	0.49	FE	0.91 (0.57–1.45)	0.69
***LRP5*** **(rs3736228)**									
Allele Contrast: T	345 (14%)/863 (13%)	2	0%	0.92	0%	0.99	FE	1.14 (0.78–1.65)	0.50
Recessive: TT+TC	195 (24%)/481 (22%)	2	0%	0.94	0%	0.89	FE	1.18 (0.78–1.77)	0.43
Homozygote Contrast: TT	195 (1%)/481 (1.6%)*	2*	N/A	N/A	N/A	N/A	N/A	N/A	N/A
***VDR*** **FokI (rs10735810)**									
Allele Contrast: C	342 (60%)/774 (48%)	3	**76%**	**0.006**	0%	0.33	RE	1.60 (0.82–3.11)	0.17
Recessive: CC+CT	220 (69%)/494 (61%)	3	**61%**	**0.05**	0%	0.36	RE	1.49 (0.76–2.91)	0.25
Homozygote Contrast: CC	220 (24%)/494 (16%)	3	0%	0.71	0%	0.86	FE	1.49 (0.98–2.26)	0.06
***VDR*** **ApaI (rs7975232)**									
Allele Contrast: C	229 (71%)/588 (70%)	2	0%	0.83	0%	0.66	FE	1.07 (0.76–1.51)	0.71
Recessive: CC+CA	152 (78%)/387 (76%)	2	0%	0.89	0%	0.91	FE	1.09 (0.68–1.73)	0.72
Homozygote Contrast: CC	152 (29%)/387 (29%)	2	11%	0.32	43%	0.19	FE	0.92 (0.60–1.41)	0.70
***VDR*** **TaqI (rs731236)**									
Allele Contrast: C	332 (48%)/776 (48%)	3	41%	0.17	28%	0.24	RE	1.05 (0.72–1.53)	0.80
Recessive: CC+CT	218 (61%)/508 (60%)	3	10%	0.34	0%	0.41	FE	1.01 (0.72–1.41)	0.96
Homozygote Contrast: CC	218 (13%)/508 (13%)	3	0%	0.52	10%	0.29	FE	0.98 (0.60–1.60)	0.95
***VDR*** **BsmI (rs1544410)**									
Allele Contrast: T	389 (62%)/2,002 (70%)	4	47%	0.09	0%	0.54	RE	0.97 (0.68–1.39)	0.87
Recessive: TT+TG	264 (72%)/1,306 (77%)	4	0%	0.50	0%	0.90	FE	1.08 (0.79–1.49)	0.62
Homozygote Contrast: TT	264 (19%)/1,306 (30%)	4	25%	0.25	0%	0.94	RE	0.85 (0.60–1.21)	0.37

RE, random effects; FE, fixed effects; OR, odds ratio; CI, confidence interval. Sample size describes frequency of case and control counts for each model with risk variant frequency in parentheses. *LRP5 (rs3736228) TT homozygotes present in only one of the two included studies. Values in bold indicate significant heterogeneity and/or associations with fracture risk.

**Table 4:** Summary effects from the overall analyses of case-control association studies for genetic variants associated with fracture occurrence risk in physically active participants including only good and moderate quality studies with sex sub-groups.

**Genetic variant and risk comparison model**	**Sample size**		**Test of Heterogeneity**				**Test of overall association**		
	**Participants**	**Studies**	**Overall**		**Between sub-groups**		**FE/RE**	**OR (95% CI)**	***P***
	**Fracture/Control (risk model frequency)**		***I*^**2**^**	***P***	***I*^**2**^**	***P***			
***COL1A1*** **Sp1 (rs1800012)**									
Allele Contrast: T	561 (21%)/1,878 (21%)	3	36%	0.20	57%	0.10	RE	0.95 (0.66–1.36)	0.77
Recessive: TT+TG	333 (33%)/1,101 (33%)	3	33%	0.22	54%	0.11	RE	0.96 (0.65–1.42)	0.84
Homozygote Contrast: TT	333 (1.8%)/1,101 (3%)	3	0%	0.53	0%	0.47	FE	0.73 (0.28–1.91)	0.53
***VDR*** **BsmI (rs1544410)**									
Allele Contrast: T	179 (68%)/1,405 (77%)	2	41%	0.18	0%	0.93	RE	0.96 (0.59–1.56)	0.87
Recessive: TT+TG	118 (75%)/915 (83%)	2	0%	0.45	0%	0.90	FE	1.00 (0.62–1.62)	1.00
Homozygote Contrast: TT	118 (27%)/915 (36%)	2	0%	0.39	0%	0.45	FE	0.93 (0.59–1.47)	0.75

*RE, random effects; FE, fixed effects; OR, odds ratio; CI, confidence interval. Sample size describes frequency of case and control counts for each model with risk variant frequency in parentheses. Values in bold indicate significant heterogeneity and/or associations with fracture risk*.

**Table 5:** Summary effects of case-control association studies for genetic variants associated with fracture occurrence risk in physically active males only.

**Genetic variant and risk comparison model**	**Sample size**		**Test of heterogeneity**		**Test of sub-group association**		
	**Participants**	**Studies**	**Within sub-group**		**FE/RE**	**OR (95% CI)**	***P***
	**Fracture/Control (risk model frequency)**		***I*^**2**^**	***P***			
***COL1A1*** **Sp1(rs1800012)**							
Allele Contrast: T	362 (17%)/1,960 (19%)	3	0%	0.99	FE	0.96 (0.70–1.31)	0.80
Recessive: TT+TG	208 (28%)/1,138 (30%)	3	0%	0.93	FE	0.97 (0.68–1.37)	0.85
Homozygote Contrast: TT	208 (2%)/1,138 (3%)	3	0%	0.43	FE	0.87 (0.28–2.70)	0.81
***CTR*** **(rs1801197)**							
Allele Contrast: T	271 (90%)/706 (89%)	2	12%	0.29	FE	1.27 (0.80–2.08)	0.30
Recessive: TT+TC	163 (92%)/442 (91%)	2	0%	0.38	FE	1.22 (0.62–2.38)	0.51
Homozygote Contrast: TT	163 (58%)/442 (51%)	2	4%	0.31	FE	1.35 (0.94–1.95)	0.11
***ESR1*** **PvuII (rs2234693)**							
Allele Contrast: C	83 (81%)/1,294 (73%)	2	0%	0.99	FE	1.53 (0.80–2.91)	0.14
Recessive: CC+CT	54 (85%)/849 (80%)	2	0%	0.96	FE	1.48 (0.69–3.20)	0.32
Homozygote Contrast: CC	54 (39%)/849 (32%)	2	48%	0.14	FE	1.32 (0.75–2.32)	0.34
***ESR1*** **XbaI (rs9340799)**							
Allele Contrast: G	85 (91%)/1,351 (87%)	2	**84%**	**0.01**	RE	0.92 (0.13–6.62)	0.96
Recessive: GG+GA	54 (93%)/849 (90%)	2	67%	0.08	RE	0.96 (0.15–6.36)	0.97
Homozygote Contrast: GG	54 (50%)/849 (44%)	2	38%	0.21	RE	0.93 (0.43–2.01)	0.86
***LRP5*** **(rs3736228)**							
Allele Contrast: T	303 (12%)/798 (12%)	2	0%	0.69	FE	1.14 (0.75–1.72)	0.54
Recessive: TT+TC	169 (21%)/443 (21%)	2	0%	0.73	FE	1.16 (0.75–1.81)	0.50
Homozygote Contrast: TT	169 (0.6%)/443 (1.1%)^*^	2^*^	N/A	N/A	N/A	N/A	N/A
***VDR*** **FokI (rs10735810)**							
Allele Contrast: C	304 (59%)/710 (49%)	3	**80%**	**0.006**	RE	1.42 (0.64–3.15)	0.39
Recessive: CC+CT	194 (68%)/454 (60%)	3	**68%**	**0.04**	RE	1.32 (0.59–2.95)	0.50
Homozygote Contrast: CC	194 (25%)/454 (17%)	3	0%	0.51	FE	1.47 (0.95–2.28)	0.09
***VDR*** **ApaI (rs7975232)**							
Allele Contrast: C	191 (71%)/531 (71%)	2	0%	0.68	FE	1.03 (0.71–1.50)	0.86
Recessive: CC+CA	127 (78%)/347 (76%)	2	0%	0.64	FE	1.08 (0.65–1.78)	0.78
Homozygote Contrast: CC	127 (28%)/347 (31%)	2	0%	0.47	FE	0.82 (0.52–1.30)	0.40
***VDR*** **TaqI (rs731236)**							
Allele Contrast: C	295 (47%)/719 (48%)	3	43%	0.17	RE	0.96 (0.65–1.44)	0.85
Recessive: CC+CT	193 (60%)/470 (60%)	3	25%	0.27	RE	0.96 (0.63–1.47)	0.85
Homozygote Contrast: CC	193 (12%)/470 (13%)	3	0%	0.70	FE	0.90 (0.54–1.52)	0.70
***VDR*** **BsmI (rs1544410)**							
Allele Contrast: T	313 (61%)/1,780 (70%)	4	**67%**	**0.03**	RE	0.89 (0.54–1.48)	0.65
Recessive: TT+TG	213 (71%)/1,162 (77%)	4	30%	0.23	RE	1.00 (0.64–1.56)	0.98
Homozygote Contrast: TT	213 (18%)/1,162 (30%)	4	47%	0.13	RE	0.84 (0.47–1.50)	0.54

RE, random effects; FE, fixed effects; OR, odds ratio; CI, confidence interval. Sample size describes frequency of case and control counts for each model with risk variant frequency in parentheses.

* *LRP5 (rs3736228) TT homozygotes present in only one of the two included studies. Values in bold indicate significant heterogeneity and/or associations with fracture risk*.

**Table 6:** Summary effects of case-control association studies for genetic variants associated with fracture occurrence risk in physically active females only.

**Genetic variant and risk comparison model**	**Sample size**		**Test of heterogeneity**		**Test of sub-group association**		
	**Participants**	**Studies**	**Within sub-group**		**FE/RE**	**OR (95% CI)**	***P***
	**Fracture/Control (risk model frequency)**		***I*^**2**^**	***P***			
***COL1A1*** **Sp1(rs1800012)**							
Allele Contrast: T	92 (15%)/242 (26%)	2	0%	0.64	FE	**0.48 (0.25–0.91)**	**0.03**
Recessive: TT+TG	52 (25%)/144 (38%)	2	0%	0.49	FE	0.51 (0.24–1.06)	0.07
Homozygote Contrast: TT	52 (2%)/144 (6%)	2	0%	0.53	FE	0.41 (0.07–2.33)	0.31
***VDR*** **BsmI (rs1544410)**							
Allele Contrast: T	76 (66%)/222 (70%)	2	0%	0.69	FE	1.13 (0.63–2.05)	0.68
Recessive: TT+TG	51 (75%)/144 (77%)	2	0%	0.90	FE	1.14 (0.52–2.48)	0.75
Homozygote Contrast: TT	51 (24%)/144 (31%)	2	6%	0.30	FE	0.87 (0.40–1.89)	0.73

*RE, random effects; FE, fixed effects; OR, odds ratio; CI, confidence interval. Sample size describes frequency of case and control counts for each model with risk variant frequency in parentheses. Values in bold indicate significant heterogeneity and/or associations with fracture risk*.

Significant overall heterogeneity was observed between studies in the *COL1A2* rs412777, *ESR1* rs9340799, and *VDR* rs10735810 meta-analyses, with significant sub-group heterogeneity found in the *COL1A1* rs1800012, *COL1A2* rs412777, and *ESR1* rs2234693 SNPs. Exclusion of poor-quality studies reduced the analysis to two genetic variants in two different genes (*COL1A1* rs1800012 and *VDR* rs1544410) from three studies, but this did not change the overall effect in these analyses.

## Discussion

The aim of this meta-analysis was to evaluate the findings of candidate gene association studies on non-osteoporotic fracture risk in physically active humans. Only ten SNPs from six different genes were independently replicated, despite the 10 studies eligible for meta-analysis including 39 SNPs from 14 different genes. A sub-analysis indicated a sex-linked significant trivial reduction of fracture risk for physically active females with the T allele of the *COL1A1* rs1800012 SNP using the allele contrast model (*p* = 0.03, *d* = −0.18). However, no statistically significant overall effect was observed from the pooled results of any SNP (*p* > 0.05).

The discordance between the results of our pooled analysis and that reported in individual studies, could be attributed to differences in methodological rigor, participant ethnicity and/or sex. The two studies that provided data for the *COL1A2* PvuII (rs412777) analysis presented conflicting results, with one reporting that the “PP” genotype halved fracture risk (Blades et al., 2010), and the other suggesting that the P allele (either PP or Pp genotype) increased fracture risk (Suuriniemi et al., 2003). In the combined analysis performed herein, these contradictory results lead to null effects, which found no significant overall effect of the *COL1A2* PvuII (rs412777) SNP with fracture risk. The results of the studies may differ if two different proximal PvuII sites in the *COL1A2* gene have been assessed, only Blades et al. (2010) report the specific reference SNP number (rs412777); or if the intervention of the study on calcium and vitamin D supplementation, from which Suuriniemi et al. (2003) recruited their participants, influenced the effect of the P allele. It should also be considered that due to the age of participants in both studies, circa 11 years, that observed genetic associations with fracture cases could have been confounded by diseases which had yet to display symptoms or be diagnosed. Nevertheless, the ethnicity of participants was not reported by Suuriniemi et al. (2003) and may have differed from the Caucasian participants studied by Blades et al. (2010). Allele frequencies and baseline risk can vary across ethnic groups and failing to account for this may result in spurious associations with candidate genes (Pérez-Lezaun et al., 1997; Thomas and Witte, 2002). The investigated SNPs included in this meta-analysis may have no functional influence on fracture risk but exist in linkage disequilibrium with other SNPs that do. These patterns of linkage disequilibrium can differ across populations and associations in one but not another may be a result of these complex differences.

Five studies investigated the genetic association of stress fracture risk in Caucasian, or predominantly Caucasian, male adult military or athletic individuals. However, five additional studies did not report participant ethnicity; three of which provided the data for the *VDR* FokI (rs10735810) SNP analysis (Chatzipapas et al., 2009; Korvala et al., 2010; Varley et al., 2018). This suggests that the C allele had no overall effect on fracture risk using the random effects meta-analysis model. However, it has been argued that random effects models are not more conservative if the relative contribution of smaller low-quality studies on the overall effect are increased (Sterne et al., 2011). A fixed effects model was not considered appropriate for the *VDR* FokI (rs10735810) analysis, as heterogeneity was significantly high (*p* = 0.006, *I*^2^ = 76%) and the participants' ethnicity unknown. Nevertheless, all three studies reported accordance with Hardy-Weinberg equilibrium and a significant trivial increase of fracture risk is associated with the C allele using a fixed effects model (*OR* = 1.37, 95% CI = 1.03–1.81, *p* = 0.03, *d* = 0.07) and ethnic variation across studies may have masked a valid genetic association.

The genetic architecture and inter-individual variation of complex traits, such as fracture risk, are determined by numerous genetic variants with a range of effect sizes, which can be very small (Gibson, 2009). Additionally, heterogeneity between genetic association studies is often high, so several replication attempts are required to determine the physiological effect of genetic variants with confidence (Salanti et al., 2005). However, the SNPs included in this meta-analysis had only been examined in two to four studies, with many authors attempting to identify novel variants, instead of examining previous findings from GWAS or other candidate gene studies. The *LRP5* and *ESR1* genes, and the *LRP5* rs3736228 SNP, have been associated with fracture risk (Trajanoska et al., 2018) or bone mineral density (Kemp et al., 2017) in GWAS. However, these studies have focused on osteoporotic fracture and/or non-athletic individuals older than 18 and GWAS on fracture risk in young physically active healthy individuals appear absent from the literature. Many of the studies included in the current meta-analysis were of poorer quality and required further verification. Nevertheless, accurate replication would be challenging, as adequate reporting of participant characteristics was a common limitation of studies. One study failed to report if Hardy-Weinberg equilibrium was observed (Välimäki et al., 2005) and three reported disequilibrium for certain SNPs which were not included in the quantitative analysis (Yanovich et al., 2012; Varley et al., 2015, 2016). Sample size was another frequently observed limitation of included studies, with only two reporting a-priori power calculations (Blades et al., 2010; Zhao et al., 2016). As the effect of genetic variants may be small, a-priori power calculations are strongly recommended in genetic association studies (Salanti et al., 2005) and several authors suggest that their studies may have been underpowered (Välimäki et al., 2005; Korvala et al., 2010; Yanovich et al., 2012; Cosman et al., 2013; Varley et al., 2016; Zhao et al., 2016). Some authors also acknowledged the potential influence that differences in nutritional status could have on bone health and thus fracture risk (Blades et al., 2010; Korvala et al., 2010; Cosman et al., 2013; Varley et al., 2018). However, none of the studies included in this meta-analysis were able to control for dietary variation between groups.

Although no overall effect of the included SNPs was observed on fracture risk in this meta-analysis some genetic variants, such as the *COL1A2* PvuII (rs412777) and *VDR* FokI (rs10735810) SNPs, may still warrant further investigation. Indeed, genetic variants in *LRP5* and *ESR1* have been associated with osteoporotic fracture risk and bone mineral density in GWAS (Kemp et al., 2017; Trajanoska et al., 2018) and genuine physiological genetic effects could have been disguised by ethnicity dependent linkage disequilibrium with other influential variants or insufficiently powered analysis. Nevertheless, none of the included SNPs currently show a significant overall effect on fracture risk in physically active male and female combined analysis and could, indeed, have no physiological influence. However, further high-quality replication attempts would provide greater clarity of the influence of genetic risk factors for fracture risk in physically active participants. In the future, researchers should ensure a-priori power calculations are conducted and reported using clearly defined homogenous sample groups to inform the understanding of potential gene-environment and gene-gene interactions.

Only one of the studies included within this meta-analysis included female only participants (Suuriniemi et al., 2003). Six included both male and female participants, of these, five reported both combined and sex specific analysis (Yanovich et al., 2012; Cosman et al., 2013; Varley et al., 2015, 2016, 2018) and one reported combined analysis only (Blades et al., 2010). Males and females are often combined in genetic association studies of injury risk, which will improve the sample size and statistical power of the analysis (Posthumus et al., 2009; Blades et al., 2010; Ficek et al., 2013). This approach is rationalized, if the genetic variants are located on the autosomal regions of the genome, by stating that the region of interest is not linked to a specific sex. However, this explanation disregards the significant differences in the relative risk of bone injuries between the sexes (Arendt et al., 1999; Renstrom et al., 2008; Wentz et al., 2011). The division of sex in this meta-analysis identified more than five times the number of male than female participants. This resulted in larger standard errors in the female sub-groups with only the *COL1A1* Sp1 (rs1800012) and *VDR* BsmI (rs1544410) SNPs replicated in females in more than one study. Stress fracture risk has been suggested to be three times greater in physically active female military personnel and 50% higher in female athletes than their male colleagues, due to biomechanical and physiological differences (Wentz et al., 2011). Indeed, the prevalence of fracture cases in the current meta-analysis was greater in females (27%) than males (21%). Significant sub-group heterogeneity was observed between sexes in the effect of the *COL1A1* Sp1 (rs1800012), *COL1A2* PvuII (rs412777), and *ESR1* PvuII (rs2234693) SNPs, highlighting the potential for sex-specific associations. Epidemiological data suggest that fracture incidence is greater in males between the age 18 and 49 than females in the general population (Curtis et al., 2016). However, the authors suggest this pattern reflects the increased prevalence of young males in high trauma events such as road traffic accidents (Curtis et al., 2016). Nevertheless, physically active females consistently demonstrate an increased risk of fracture when compared to their male counterparts (Kaufman et al., 2000; Wentz et al., 2011; Waterman et al., 2016). Therefore, the relative contribution of genetic susceptibility to fracture risk, and potential of preventative strategies, is likely to be greater in physically active females than males.

The *COL1A1* Sp1 (rs1800012) SNP, located in the 1st intron of the *COL1A1* gene, is one of the most extensively investigated genetic variants in the injury risk literature. Sub-group analysis within the current meta-analysis indicated a trivial reduction in fracture risk for the T allele of the *COL1A1* rs1800012 SNP in physically active females, but not males. However, the T allele was not associated with fracture risk when males and females were combined (Blades et al., 2010; Cosman et al., 2013; Varley et al., 2018), nor in males only (Korvala et al., 2010). The observed reduction in fracture risk associated with the T allele in females was not independently reported by the two studies included, which provided data for this meta-analysis (Cosman et al., 2013; Varley et al., 2018). Whilst it should be acknowledged that despite pooling data from these two studies this finding is only based on 196 females in total the T allele has been consistently associated with increased risk of osteoporotic fracture due to reduced bone mineral density in elderly post-menopausal females (Mann et al., 2001; Mann and Ralston, 2003). Nevertheless, the T allele has been repeatedly associated with reduced ligament injury risk in physically active mixed sex (Khoschnau et al., 2008; Posthumus et al., 2009; Ficek et al., 2013) and male participants (Stepien-Słodkowska et al., 2013). These previous findings in addition to those of the current meta-analysis suggest the T allele could be associated with protection against some sport and exercise related injuries but further research is still required.

The T allele of the rs1800012 *COL1A1* SNP is associated with greater Sp1 binding affinity and COL1A1 production, which is similar between male and female carriers (Mann et al., 2001). This results in an increased relative abundance of type 1 procollagen formed exclusively from COL1A1 polypeptides, which has been suggested to be weaker than the normal COL1A1/ COL1A2 combination (Mann et al., 2001). However, this is based on the increased osteoporotic fracture risk associated with the T allele in the elderly and is contradicted by the protective effect of the T allele observed in this meta-analysis and other studies of sport and exercise related ligament injuries (Khoschnau et al., 2008; Posthumus et al., 2009; Ficek et al., 2013; Stepien-Słodkowska et al., 2013). Participants included in this meta-analysis and in the injury risk literature include physically active individuals, who are much younger than those studied in association with osteoporotic fracture. Mechanical loading of the musculoskeletal system is increased during sport and physical activity (Launay, 2015; Meardon et al., 2015; Bacon and Mauger, 2017; Schuh-Renner et al., 2017). Therefore, the T allele may result in mechanically stronger type 1 collagen, which is protective against ligamentous and bone injuries at younger ages in physically active individuals. The T allele may increase osteoporosis susceptibility in the elderly due to other pathogenic factors, such as excessive bone resorption. Alternatively, differences in the mechanical properties of bone and ligament may explain the observed variations in injury susceptibility and further investigation of the influence of the T allele on fracture risk in young physically active participants is needed.

Genetic variants do not necessarily result in dichotomous injured or non-injured states and genetic penetrance describes the probability that a carrier of a risk allele will express the disease/injury trait (Cooper et al., 2013). The genetic penetrance of the *COL1A1* rs1800012 SNP with fracture risk may be sex-specific and influenced by age. Therefore, it is possible that the T allele of the *COL1A1* rs1800012 SNP is concurrently associated with a reduced risk of bone fracture in young physically active females and an increased risk of osteoporotic fracture in elderly females. This finding is based on a total of 204 female participants (53 fractures and 151 controls), 10 of which were TT homozygotes. This is lower than expected (~5%) considering the overall size of the sample as the minor T allele is present in ~16–19% of Europeans and 9–13% of individuals globally. It may be that no association was observed in the recessive and homozygote contrast models or the individual studies (Cosman et al., 2013; Varley et al., 2018) as the number of T homozygotes, and overall participants, was low. Pooling the results of multiple genetic association studies becomes highly valuable to improve the statistical power of the analysis but the effect of the *COL1A1* rs1800012 SNP T allele on fracture risk should be replicated in a large group of physically active females in order to examine the finding of this meta-analysis.

The aim of the current meta-analysis was to synthesize the findings and quality of genetic case-control association studies on fracture risk in physically active participants. Sex-specific analysis indicated a protective effect of the *COL1A1* (rs1800012) T allele in females despite previous associations with increased risk of osteoporotic fracture in the elderly. This suggests that the genetic penetrance of the T allele is influenced by sex/age and is not ubiquitously detrimental to bone strength as has been previously suggested. The null effects observed in the overall analyses of SNPs included in this meta-analysis should not be considered finite due to potential limitations of the included studies. Pediatric participants, only present in the *COL1A1* (rs1800012) and *COL1A2* PvuII (rs412777) combined sex analyses, are more likely to include individuals with undiagnosed asymptomatic diseases which could influence the genetic association results. Nevertheless, the overall findings *COL1A1* (rs1800012) combined sex analyses do not change if pediatric participants are removed. However, the *COL1A2* PvuII (rs412777) analyses is comprised exclusively of pediatric participants and should, therefore, be considered specific to this population and with the limitations discussed. Readers should also consider the potential influence that nutritional differences which could interact with the exposure of physical activity and genetic predisposition to mediate susceptibility to fracture occurrence in the included studies. Overall review of study designs indicated several recommendations for consideration in future research such as the inclusion of a-priori power calculations, sex-specific analysis, and greater clarity in the reporting of participant ethnicity. Consequently, further high-quality investigation of the *COL1A1* (rs1800012), *COL1A2* PvuII (rs412777), and *VDR* FokI (rs10735810) SNPs with fracture risk in a homogenous sample of physically active participants is warranted.

## Data Availability Statement

The raw data supporting the conclusions of this article will be made available by the authors, without undue reservation, to any qualified researcher.

## Author Contributions

ER-M performed the initial literature search and eligibility screening which was replicated by MW. ER-M and YM independently completed the quality and risk of bias assessments for eligible studies. Data extraction and analysis were performed by ER-M, YM, and MW. The first draft of the manuscript was written by ER-M. Discrepancies were resolved by consensus agreement amongst all authors. All authors commented on previous versions of the manuscript, approved the final manuscript, and contributed to the study conception and design.

## Conflict of Interest

Sponsorship to complete the research was provided by St Mary's University, Twickenham and Fulham Football Club, London as part of ER-M's. The authors acknowledge the potential conflict of interest resulting from YM's role as Head of Nutrigenetics for Nell, a well-being company that incorporates genetics analyses in its approach. Nevertheless, the research was conducted in a fair, honest, impartial, and transparent manner. The remaining author declares that the research was conducted in the absence of any commercial or financial relationships that could be construed as a potential conflict of interest.

## References

[B1] Andrew T. Leto A. Scurrah K. J. MacGregor A. J. Spector T. D. (2004). Risk of wrist fracture in women is heritable and is influenced by genes that are largely independent of those influencing BMD. J. Bone Mineral Res. 20, 67–74. 10.1359/JBMR.041015 15619671

[B2] Arendt E. A. Agel J. Dick R. (1999). Anterior cruciate ligament injury patterns among collegiate men and women. J. Athletic Train. 34, 86–92. 16558564 PMC1322895

[B3] Bacon C. Mauger S. A. R. (2017). Prediction of overuse injuries in professional U18-U21 footballers using metrics of training distance and intensity. J. Strength Cond. Res. 31, 3067–3076. 10.1519/JSC.0000000000001744 27930446

[B4] Baumert P. Lake M. Stewart J. Drust C. E. Erskine B. Lake R. M.. (2016). Genetic variation and exercise-induced muscle damage: implications for athletic performance, injury and ageing. Eur. J. Appl. Physiol. 116, 1595–1625. 10.1007/s00421-016-3411-1 27294501 PMC4983298

[B5] Bennell K. Matheson G. Meeuwisse W. Brukner P. (1999). Risk factors for stress fractures. Sports Med. 28, 1–32. 10.2165/00007256-199928020-00004 10492029

[B6] Blades H. Z. Arundel P. Carlino W. A. Dalton A. Crook J. S. Freeman J. V.. (2010). Collagen gene polymorphisms influence fracture risk and bone mass acquisition during childhood and adolescent growth. Bone 47, 989–994. 10.1016/j.bone.2010.08.014 20736093

[B7] Chatzipapas C. Boikos S. Drosos G. I. Kazakos K. Tripsianis G. Serbis A.. (2009). Polymorphisms of the vitamin D receptor gene and stress fractures. Hormone Metabol. Res. 41, 635–640. 10.1055/s-0029-1216375 19391078 PMC3135021

[B8] Cohen J. (1988). Statistical Power Analysis for the Behavioral Sciences, 2nd Edn. New York, NY: Routledge.

[B9] Conn J. M. Annest J. L. Gilchrist J. (2003). Sports and recreation related injury episodes in the US population, 1997–99. Injury Prev. 9, 117–123. 10.1136/ip.9.2.117 12810736 PMC1730974

[B10] Cooper D. N. Krawczak M. Polychronakos C. Tyler-Smith C. Kehrer-Sawatzki H. (2013). Where genotype is not predictive of phenotype: towards an understanding of the molecular basis of reduced penetrance in human inherited disease. Hum. Gene. 132, 1077–1130. 10.1007/s00439-013-1331-2 23820649 PMC3778950

[B11] Cosman F. Ruffing J. Zion M. Uhorchak J. Ralston S. Tendy S.. (2013). Determinants of stress fracture risk in United States military academy cadets. Bone 55, 359–366. 10.1016/j.bone.2013.04.011 23624291

[B12] Curtis E. M. van der Velde R. Moon R. J. van den Bergh J. P. W. Geusens P. de Vries F.. (2016). Epidemiology of fractures in the United Kingdom 1988-2012: variation with age, sex, geography, ethnicity and socioeconomic status. Bone 87, 19–26. 10.1016/j.bone.2016.03.006 26968752 PMC4890652

[B13] DeLuca H. (2005). Overview of general physiologic features and functions of vitamin D1-4. Am. J. Clin. Nutr. 80, 1689S–1696S. 10.1093/ajcn/80.6.1689S 15585789

[B14] Efstathiadou Z. Tsatsoulis A. Ioannidis J. P. (2001). Association of collagen ialpha 1 Sp1 polymorphism with the risk of prevalent fractures: a meta-analysis. J. Bone Mineral Res. 16, 1586–1592. 10.1359/jbmr.2001.16.9.1586 11547828

[B15] Egger M. Smith G. D. Schneider M. Minder C. (1997). Bias in meta-analysis detected by a simple, graphical test. BMJ Clin. Res. Ed. 315:629. 10.1136/bmj.315.7109.629 9310563 PMC2127453

[B16] Ficek K. Cieszczyk P. Kaczmarczyk M. Maciejewska-Karłowska A. Sawczuk M. Cholewinski J.. (2013). Gene variants within the COL1A1 gene are associated with reduced anterior cruciate ligament injury in professional soccer players. J. Sci. Med. Sport 16, 396–400. 10.1016/j.jsams.2012.10.004 23168334

[B17] Gibson W. T. (2009). Genetic association studies for complex traits: relevance for the sports medicine practitioner. Br. J. Sports Med. 43, 314–316. 10.1136/bjsm.2008.052191 19066182

[B18] Hägglund M. Waldén M. Magnusson H. Kristenson K. Bengtsson H. Ekstrand J. (2013). Injuries affect team performance negatively in professional football: an 11-year follow-up of the UEFA champions league injury study. Br. J. Sports Med. 47, 738–742. 10.1136/bjsports-2013-092215 23645832

[B19] Herbert A. J. Williams A. G. Hennis P. Erskine J. Sale R. M. Day C.. (2018). The interactions of physical activity, exercise and genetics and their associations with bone mineral density: implications for injury risk in elite athletes. Eur. J. Appl. Physiol. 119, 29–47. 10.1007/s00421-018-4007-8 30377780 PMC6342881

[B20] Ioannidis J. P. A. Trikalinos T. A. Ntzani E. E. Contopoulos-Ioannidis D. G. (2001). Replication validity of genetic association studies. Nat. Genet. 29, 306–309. 10.1038/ng749 11600885

[B21] Ioannidis J. P. A. Trikalinos T. A. Ntzani E. E. Contopoulos-Ioannidis D. G. (2003). Genetic associations in large versus small studies: an empirical assessment. Lancet 361, 567–571. 10.1016/S0140-6736(03)12516-0 12598142

[B22] Ji G.-R. Yao M. Sun C.-Y. Li Z.-H. Han Z. (2010). Bsm I, Taq I, ApaI and FokI polymorphisms in the vitamin D receptor (VDR) gene and risk of fracture in caucasians: a meta-analysis. Bone 47, 681–686. 10.1016/j.bone.2010.06.024 20601302

[B23] Kaufman K. Brodine S. Shaffer R. (2000). Military training-related injuries Surveillance, research, and prevention. Am. J. Prev. Med. 18, 54–63. 10.1016/S0749-3797(00)00114-8 10736541

[B24] Kemp J. P. Morris J. A. Medina-Gomez C. Forgetta V. Warrington N. Youlten M.. (2017). Identification of 153 new loci associated with heel bone mineral density and functional involvement of GPC6 in osteoporosis. Nat. Gene. 49, 1468–1475. 10.1038/ng.3949 28869591 PMC5621629

[B25] Khoschnau S. MeIhus H. Jacobson A. Rahme H. Bengtsson H. Ribom E. (2008). Type I Collagen A1 Sp1 polymorphism and the risk of cruciate ligament ruptures or shoulder dislocations. Am. J. Sports Med. 36, 2432–2436. 10.1177/0363546508320805 18669982

[B26] Kohrt W. M. Bloomfield S. A. Little K. D. Nelson M. E. Yingling V. R. (2004). Physical activity and bone health. Med. Sci. Sports Exercise 36, 1985–1996. 10.1249/01.MSS.0000142662.21767.58 15514517

[B27] Korvala J. Hartikka H. Pihlajamäki H. Solovieva S. Ruohola J.-P. Sahi T.. (2010). Genetic predisposition for femoral neck stress fractures in military conscripts. BMC Gene. 11:95. 10.1186/1471-2156-11-95 20961463 PMC2975640

[B28] Kozlovskaia M. Vlahovich N. Ashton K. J. Hughes D. C. (2017). Biomedical risk factors of achilles tendinopathy in physically active people: a systematic review. Sports Med. Open 3:20. 10.1186/s40798-017-0087-y 28523640 PMC5436990

[B29] Launay F. (2015). Sports-related overuse injuries in children. Ortho. Traumatol. Surg. Res. 101, S139–S147. 10.1016/j.otsr.2014.06.030 25555804

[B30] Le Gall F. Carling C. Reilly T. Vandewalle H. Church J. Rochcongar P. (2006). Incidence of injuries in elite french youth soccer players: a 10-season study. Am. J. Sports Med. 34, 928–938. 10.1177/0363546505283271 16436535

[B31] Lee Y. H. (2015). Meta-analysis of genetic association studies. Ann. Lab. Med. 35, 283–287. 10.3343/alm.2015.35.3.283 25932435 PMC4390695

[B32] Mann V. Hobson E. E. Li B. Stewart T. L. Grant S. F. Robins S. P.. (2001). A COL1A1 sp1 binding site polymorphism predisposes to osteoporotic fracture by affecting bone density and quality. J. Clin. Invest. 107, 899–907. 10.1172/JCI10347 11285309 PMC199568

[B33] Mann V. Ralston S. H. (2003). Meta-analysis of COL1A1 sp1 polymorphism in relation to bone mineral density and osteoporotic fracture. Bone 32, 711–717. 10.1016/S8756-3282(03)00087-5 12810179

[B34] Meardon S. A. Willson J. D. Gries S. R. Kernozek W. T. Derrick T. R. (2015). Bone stress in runners with tibial stress fracture. Clin. Biomechan. 30, 895–902. 10.1016/j.clinbiomech.2015.07.012 26282463

[B35] Meeuwisse W. H. Tyreman H. Hagel B. Emery C. (2007). A dynamic model of etiology in sport injury: the recursive nature of risk and causation. Clin. J. Sport Med. 17, 215–219. 10.1097/JSM.0b013e3180592a48 17513916

[B36] Michaëlsson K. Melhus H. Ferm H. Ahlbom A. Pedersen N. L. (2005). Genetic liability to fractures in the elderly. Arch. Inter. Med. 165:1825. 10.1001/archinte.165.16.1825 16157825

[B37] Moher D. Liberati A. Tetzlaff J. Altman D. G. (2009). Preferred reporting items for systematic reviews and meta-analyses: the PRISMA statement. PLoS Med. 6:e1000097 10.1371/journal.pmed.1000097 19621072 PMC2707599

[B38] Pérez-Lezaun A. Calafell F. Mateu E. Comas D. Bosch E. Bertranpetit J. (1997). Allele frequencies for 20 microsatellites in a worldwide population survey. Hum. Heredity 47, 189–196. 10.1159/000154412 9239505

[B39] Posthumus M. September A. V. Keegan M. O'Cuinneagain D. Van der Merwe W. Schwellnus M.. (2009). Genetic risk factors for anterior cruciate ligament ruptures: COL1A1 gene variant. Br. J. Sports Med. 43, 352–356. 10.1136/bjsm.2008.056150 19193663

[B40] Qasim A. Mayhew A. J. Ehtesham S. Alyass A. Volckmar A.- L. Herpertz S.. (2019). Gain-of-function variants in the melanocortin 4 receptor gene confer susceptibility to binge eating disorder in subjects with obesity: a systematic review and meta-analysis. Obes. Rev. 20, 13–21. 10.1111/obr.12761 30306707

[B41] Renstrom P. Ljungqvist A. Arendt E. Beynnon B. Fukubayashi T. Garrett W.. (2008). Non-contact ACL injuries in female athletes: an international olympic committee current concepts statement. Br. J. Sports Med. 42, 394–412. 10.1136/bjsm.2008.048934 18539658 PMC3920910

[B42] Salanti G. Sanderson S. Higgins J. P. T. (2005). Obstacles and opportunities in meta-analysis of genetic association studies. Gene. Med. 7, 13–20. 10.1097/01.GIM.0000151839.12032.1A 15654223

[B43] Sánchez-Meca J. Marín-Martínez F. Chacón-Moscoso S. (2003). Effect-size indices for dichotomized outcomes in meta-analysis. Psychol. Methods 8, 448–467. 10.1037/1082-989X.8.4.448 14664682

[B44] Schuh-Renner A. Grier T. L. Canham-Chervak M. Hauschild V. D. Roy T. C. Fletcher J.. (2017). Risk factors for injury associated with low, moderate, and high mileage road marching in a U.S. army infantry brigade. J. Sci. Med. Sport 20:S28–33. 10.1016/j.jsams.2017.07.027 28986087

[B45] Sohani Z. N. Meyre D. de Souza R. J. Joseph P. G. Gandhi M. Dennis B. B.. (2015). Assessing the quality of published genetic association studies in meta-analyses: the quality of genetic studies (Q-Genie) tool. BMC Gene. 16:50. 10.1186/s12863-015-0211-2 25975208 PMC4431044

[B46] Stepien-Słodkowska M. Ficek K. Eider J. Leonska-Duniec A. Maciejewska-Karłowska A. Sawczuk M.. (2013). The +1245g/t polymorphisms in the collagen type i alpha 1 (col1a1) gene in polish skiers with anterior cruciate ligament injury. Biol. Sport 30, 57–60. 10.5604/20831862.1029823 24744467 PMC3944561

[B47] Sterne J. A. Hernán M. A. Reeves B. C. Savović J. Berkman N. D. Viswanathan M.. (2016). ROBINS-I: A tool for assessing risk of bias in non-randomised studies of interventions. BMJ Clin. Res. Ed. 355:i4919. 10.1136/bmj.i4919 27733354 PMC5062054

[B48] Sterne J. A. Sutton A. J. Ioannidis J. P. Terrin N. Jones D. R. Lau J.. (2011). Recommendations for examining and interpreting funnel plot asymmetry in meta-analyses of randomised controlled trials. BMJ 343:343. 10.1136/bmj.d4002 21784880

[B49] Suuriniemi M. Kovanen V. Mahonen A. Alén M. Wang Q. Lyytikäinen A.. (2006). COL1A1 Sp1 polymorphism associates with bone density in early puberty. Bone 39, 591–597. 10.1016/j.bone.2006.02.053 16580273

[B50] Suuriniemi M. Mahonen A. Kovanen V. Alén M. Cheng S. (2003). Relation of Pvu II site polymorphism in the COL1A2 gene to the risk of fractures in prepubertal finnish girls. Physiol. Genom. 14, 217–224. 10.1152/physiolgenomics.00070.2003 12813128

[B51] Thomas D. C. Witte J. S. (2002). Point: population stratification: a problem for case-control studies of candidate-gene associations? Cancer Epidemiol. Biomark. Prev. 11, 505–512. Available online at: https://cebp.aacrjournals.org/content/11/6/505.full#related-urls 12050090

[B52] Thompson P. D. Arena R. Riebe D. Pescatello L. S. (2013). ACSM's new preparticipation health screening recommendations from ACSM's guidelines for exercise testing and prescription, ninth edition. Curr. Sports Med. Rep. 12, 215–217. 10.1249/JSR.0b013e31829a68cf 23851406

[B53] Trajanoska K. Morris J. A. Oei L. Zheng H.-F. Evans D. Kiel M.. (2018). Assessment of the genetic and clinical determinants of fracture risk: genome wide association and mendelian randomisation study. BMJ 362:k3225. 10.1136/bmj.k3225 30158200 PMC6113773

[B54] Välimäki V.-V. Alfthan H. Lehmuskallio E. Löyttyniemi E. Sahi T. Suominen H.. (2005). Risk factors for clinical stress fractures in male military recruits: a prospective cohort study. Bone 37, 267–273. 10.1016/j.bone.2005.04.016 15964254

[B55] Varley I. Greeves J. P. Sale C. Friedman E. Moran D. S. Yanovich R.. (2016). Functional polymorphisms in the P2X7 receptor gene are associated with stress fracture injury. Purinergic Signal. 12, 103–113. 10.1007/s11302-016-9495-6 26825304 PMC4749527

[B56] Varley I. Hughes D. Greeves C. Stellingwerff J. P. Ranson T. Fraser C.. (2015). RANK/RANKL/OPG pathway: genetic associations with stress fracture period prevalence in elite athletes. Bone 71, 131–136. 10.1016/j.bone.2014.10.004 25464125

[B57] Varley I. Hughes D. Greeves C. Stellingwerff J. P. T. Ranson C. Fraser W.. (2018). The association of novel polymorphisms with stress fracture injury in elite athletes: further insights from the SFEA cohort. J. Sci. Sport 21, 564–568. 10.1016/j.jsams.2017.10.038 29129460

[B58] Waterman B. R. Gun B. Bader J. Orr O. J. D Belmont P. J. (2016). Epidemiology of lower extremity stress fractures in the United States military. Military Med. 181, 1308–1313. 10.7205/MILMED-D-15-00571 27753569

[B59] Wentz L. Liu P. Y. Haymes E. Ilich J. Z. (2011). Females have a greater incidence of stress fractures than males in both military and athletic populations: a systemic review. Military Med. 176, 420–430. 10.7205/MILMED-D-10-00322 21539165

[B60] Yanovich R. Friedman E. Milgrom R. Oberman B. Freedman L. Moran D. S. (2012). Candidate gene analysis in israeli soldiers with stress fractures. J. Sports Sci. Med. 11, 147–155. Available online at: https://www.ncbi.nlm.nih.gov/pmc/articles/PMC3737837/24149131 PMC3737837

[B61] Zhao L. Chang Q. Huang T. Huang C. (2016). Prospective cohort study of the risk factors for stress fractures in chinese male infantry recruits. J. Int. Med. Res. 44, 787–795. 10.1177/0300060516639751 27207942 PMC5536631

